# A Gel-Based Proteomic Analysis Reveals Synovial α-Enolase and Fibrinogen β-Chain Dysregulation in Knee Osteoarthritis: A Controlled Trial

**DOI:** 10.3390/jpm13060916

**Published:** 2023-05-30

**Authors:** Maria Teresa Rocchetti, Davide Bizzoca, Lorenzo Moretti, Enrico Ragni, Francesco Luca Moretti, Giovanni Vicenti, Giuseppe Solarino, Alessandro Rizzello, Vittoria Petruzzella, Luigi Leonardo Palese, Salvatore Scacco, Giuseppe Banfi, Biagio Moretti, Antonio Gnoni

**Affiliations:** 1 Department of Clinical and Experimental Medicine, University of Foggia, Via Pinto 1, 71122 Foggia, Italy; mariateresa.rocchetti@unifg.it; 2 Orthopaedics Unit—UOSD Vertebral Surgery, DAI Neuroscience, Sense Organs and Locomotor System, AOU Consorziale Policlinico, 70124 Bari, Italygiovanni.vicenti@uniba.it (G.V.); giuseppe.solarino@uniba.it (G.S.);; 3 PhD Course in Public Health, Clinical Medicine and Oncology, DiMePre-J, University of Bari “Aldo Moro”, Piazza Giulio Cesare 11, 70124 Bari, Italy; 4 IRCCS Istituto Ortopedico Galeazzi, Laboratorio di Biotecnologie Applicate all’Ortopedia, Via R. Galeazzi 4, 20161 Milano, Italy; enrico.ragni@grupposandonato.it (E.R.);; 5 National Centre for Chemicals, Cosmetic Products and Consumer Protection, National Institute of Health, 00161 Rome, Italy; 6 Clinical Biochemistry, DiBraiN, School of Medicine, University of Bari “Aldo Moro”, 70124 Bari, Italy; a.rizzello20@studenti.uniba.it (A.R.); salvatore.scacco@uniba.it (S.S.);; 7 IRCCS Galeazzi-Sant’Ambrogio, Via Cristina Belgioioso 173, 20157 Milano, Italy; 8 Faculty of Medicine, Università Vita-Salute San Raffaele, Via Olgettina 58, 20132 Milano, Italy

**Keywords:** synovial fluid, proteome, precision medicine, knee osteoarthritis, meniscal tears, gel-based approach

## Abstract

Background: The identification of synovial fluid (SF) biomarkers that could anticipate the diagnosis of osteoarthritis (OA) is gaining increasing importance in orthopaedic clinical practice. This controlled trial aims to assess the differences between the SF proteome of patients affected by severe OA undergoing Total Knee Replacement (TKR) compared to control subjects (i.e., subjects younger than 35, undergoing knee arthroscopy for acute meniscus injury). Methods: The synovial samples were collected from patients with Kellgren Lawrence grade 3 and 4 knee osteoarthritis undergoing THR (study group) and young patients with meniscal tears and no OA signs undergoing arthroscopic surgery (control group). The samples were processed and analyzed following the protocol defined in our previous study. All of the patients underwent clinical evaluation using the International Knee Documentation Committee (IKDC) subjective knee evaluation (main outcome), Knee Society Clinical Rating System (KSS), Knee injury and Osteoarthritis Outcome Score (KOOS), and Visual Analogue Scale (VAS) for pain. The drugs’ assumptions and comorbidities were recorded. All patients underwent preoperative serial blood tests, including complete blood count and C-Reactive Protein (CRP). Results: The synovial samples’ analysis showed a significantly different fibrinogen beta chain (FBG) and alpha-enolase 1 (ENO1) concentration in OA compared to the control samples. A significant correlation between clinical scores, FBG, and ENO1 concentration was observed in osteoarthritic patients. Conclusions: Synovial fluid FBG and ENO1 concentrations are significantly different in patients affected by knee OA compared with non-OA subjects.

## 1. Introduction

Osteoarthritis (OA) is a highly prevalent degenerative joint disease and the most common cause of pain and disability in elderly people. The Osteoarthritis Research Society International (OARSI) defines OA as “a disorder involving movable joints characterized by cell stress and extracellular matrix degradation initiated by micro- and macro-injury that activates maladaptive repair responses including pro-inflammatory pathways of innate immunity” [1].

OA affects 250 million individuals worldwide, mainly adults over 65, and causes significant social health problems [2].

Even if several genetic and environmental risk factors promote the development of OA, i.e., gender, age, body mass index (BMI), physical activity, ethnicity, and muscle weakness, the exact pathogenesis of OA is still unclear [3]. The anatomopathological features of OA include intra-articular synovial inflammation, cartilage degeneration, meniscal degeneration, subchondral osteosclerosis, and inflammation and fibrosis of the infrapatellar fat pad [4].

Currently, the diagnosis of OA is based on physical examination, while the progression of the joint degenerative changes can only be assessed with imaging techniques [3,4].

Osteoarthritis causes joint stiffness, articular pain, and loss of function, thus, progressively affecting the patient’s social life and working ability [5,6,7,8]. Nonetheless, the onset of the symptoms occurs when irreversible articular changes have developed [2]. Therefore, arthroplasty still is the most successful procedure for the treatment of OA, with a reported patient satisfaction rate [9].

Consequently, the identification of synovial fluid (SF) biomarkers that could predict the diagnosis of OA before the onset of the symptoms is gaining increasing importance in orthopaedics [2]. In this context, the analysis of synovial fluid by proteomics may play a central role in the future.

The proteome is the organism’s specific proteins produced in a defined physiological context at a specific point in time [7]. Proteome analysis or “proteomics” is the large-scale systematic analysis of the proteome from a cell, tissue, or entire organism [7]. It is a relatively new discipline that has been growing rapidly in the last two decades [8].

Proteomics of the SF in OA patients has depicted the upregulation of several components of the classic complement pathway and has also suggested some pro-inflammatory cytokines, namely IL-6, IL-8, and IL-18, could have a role in OA pathogenesis [6,7,8,9,10,11,12,13,14,15].

Even if previous studies have investigated the SF fluid proteome in OA [7,8,9,10,11,12,13,14,15], the application of their results is difficult and limited, mainly because of the lack of standardization in the pre-analytical phase. The main limits of these studies include the use of different animal models, the lack of reproducible study and control groups, and the use of different strategies for albumin and IgGs depletion in sample preparation for proteome analysis. These concerns have been overcome in our previous work [16].

This study compares the synovial fluid proteome of patients affected by severe OA undergoing Total Knee Replacement (TKR) to control patients (i.e., patients younger than 35, undergoing knee arthroscopy for acute meniscus injury).

## 2. Materials and Methods

### 2.1. Patients Selection

SF samples from patients with OA were prospectively collected at our Orthopaedic and Trauma Unit, between January 2019 and January 2020, from patients who underwent total knee arthroplasty (TKA) for tricompartimental knee OA. OA diagnosis was performed by an experienced Orthopaedic surgeon, following the classification criteria of the American College of Rheumatology (ACR) for knee OA [16].

Ethical approval was obtained from our centre’s ethics committee according to the 1964 Declaration of Helsinki (code: SINOVIALE-20), and all patients signed informed consent before to be enabling in the study.

Inclusion criteria: moderate-severe tricompartimental knee OA, grade 3 or 4 according to Kellgren-Lawrence (KL) classification; age between 50 and 80 years old; gender: both. Exclusion criteria: diabetes mellitus; coagulopathies; rheumatoid arthritis or other autoimmune arthritis; chronic inflammatory disease; previous surgery of the affected knee; previous intra-articular injections in the last 12 months.

In the same period, SF samples collected from patients who underwent knee arthroscopy for acute traumatic meniscal injury were used as controls. Exclusion criteria: diabetes mellitus; coagulopathies; rheumatoid arthritis or other autoimmune arthritis; chronic inflammatory diseases; previous surgery of the affected knee; degenerative meniscal injuries; previous intra-articular injections in the last 12 months; osteoarthritis; obesity; history of joint infection.

All of the patients underwent a preoperative clinical, radiographic, and laboratory evaluation by the current clinical practice standards. Preoperative X-rays included knee anteroposterior and lateral views, axial view of the patella, and full-length hip-to-ankle upstanding anteroposterior radiographs. At enrollment, anthropometric data, osteoarthritis stage according to KL classification, Knee Society Clinical Rating System (KSS) [17], International Knee Documentation Committee (IKDC) [18] subjective knee evaluation (main outcome), Knee injury and Osteoarthritis Outcome Score (KOOS) [19], and Visual Analogue Scale (VAS) for pain, drugs assumption, and comorbidities were registered. All of the patients underwent preoperatively complete blood counts, including a C-Reactive Protein (CRP) test.

### 2.2. Synovial Fluid Sample Collection and Processing

Synovial fluid sample collection was carried out as previously described [16]. The samples without detectable hemolysis were centrifuged at 400× *g* at 4 °C for 5 min to remove cells and debris, then aliquoted and stored at −80 °C until use.

A five hundred μL aliquoted sample was thawed and treated with 1 μg/mL hyaluronidase (Sigma-Aldrich, Gillingham, UK) at 37 °C for 1 h [16], then centrifuged at 10,000× *g*, at 5 °C for 10 min. The Bradford method measured the total SF protein concentration by eluding a mean of 13 μg/μL (±1.4).

The SF samples treated with 1 μg/mL hyaluronidase were concentrated by 3-kDa cut-off Amicon filter (Millipore, Billerica, MA, USA) to reach the concentration of about 25 μg/μL and treated with ProteoMiner™ Small Capacity beads (Bio-Rad Laboratories, Hercules, CA, USA). Five mg of SF’s proteome were appropriately adapted to its subcolumn and processed according to the manufacturer’s instructions. Finally, the enriched SF protein concentration was determined.

### 2.3. Two-Dimensional Gel Electrophoresis (2DE)

Enriched SF proteins were analyzed by 2D-PAGE as previously described [20]. Briefly, analytical gel 40 μg of SF proteins was isoelectrofocused (IEF) on Immobiline IPG strips (7 cm, pH 3–10 nonlinear gradient), which were previously rehydrated. IPG strips were focused on a total of 22 kVh produced by overnight run (PROTEAN IEF cell, Bio-Rad) and stored at −80 °C.

Six pools of four OA SF samples were used for 2D-PAGE, which was run in triplicate. The synovial fluids were pooled before concentration by 3-kDa cut-off Amicon filter devices to reach the suitable concentration (about 25 μg/μL) for ProteoMiner™ Small Capacity bead treatments. Control synovial fluids samples were run as described by Bizzoca et al. [20].

The second-dimension run was carried out using NuPAGE™ 4–12% Bis-Tris ZOOM™ Protein Gel after strips equilibration using NuPAGE™ MES SDS Running Buffer. SYPRO^®^ Ruby was used to staining the analytical gels, which were acquired by a scanner (PROXPRESS 2D) [21]. Image analysis of analytical 2D gels was carried out by using Image Master Platinum 2D software [21]. The coefficient of variation (CV) was used to express the precision of 2DE analyses; it is defined as the ratio of the standard deviation to the mean value.

Preparative gels were run 300 micrograms of proteins on IPGstrips, stained with QC Colloidal Blue Coomassie, and used for protein spot excision to allow mass spectrometry protein identification. For details on the solvents used, see Appendix A.

### 2.4. Mass Spectrometry Analysis and Bioinformatic Analysis

The selected protein spots were trypsin digested and analysed by MALDI-TOF-MS instrument (Autoflex III™ TOF/TOF200, Bruker Daltonics, Bremen Germany) as previously reported [21]. Protein identification was conducted against the Swissprot databases by using a database search (Biotools 3.2) and a search algorithm (MASCOT, Boston, USA, http://www.matrixscience.com accessed on 10 February 2023)). For the database search, the following parameters were used: taxonomy: human; enzyme: trypsin; fixed modification: carbamidomethyl for cysteine residues; variable modification: oxidation of methionine; one missing cleavage; and 100 ppm as mass tolerance (monoisotopic peptide masses).

A MASCOT protein score of 56 for Peptide Mass Fingerprinting (*p* < 0.05) identified the protein. Identified proteins are reported in Table 1. The protein-protein interaction network has been determined by BioGrid (https://thebiogrid.org/849780, accessed on 15 January 2023). ENO1 interacts with 519 different proteins, as reported in Figure 2, left panel. Focusing on those interactions, particular attention should be addressed to the following proteins: the ubiquitin-like modifier ISG15 [22,23]; triosephosphate isomerase 1 (TPI1) [24]; Trafficking Protein Particle Complex Subunit 2 (TRAPPC2), which could lead to premature hip osteoarthritis, often requiring total hip replacement [25]; and Tyrosine 3-Monooxygenase/Tryptophan 5-Monooxygenase Activation Protein Zeta (YWHAZ), which has been considered as a candidate housekeeping gene in the synovium osteoarthritic knees [26].

### 2.5. Statistical Analysis

Mean ± SD has been used to express the quantitative variables. To test statistically (*p*-values ≤ 0.05), the differences between quantitative variables by Mann–Whitney U-test were used, as appropriate. After the Shapiro-Wilk test, the Pearson correlation test was performed to assess any relationship between synovial biomarkers, serum biomarkers, and clinical findings (KOOS, KSS, IKDC, VAS).

Statistical analysis was performed using the Statview software package (Version 5.0, SAS Institute Inc., Cary, NC, USA).

## 3. Results and Discussion

### 3.1. Results

Table 1 summarizes the main clinical and demographic data of the enrolled OA and control patients. Twenty-four patients affected by knee OA (male: 11; female: 13; mean age: 68.19 ± 6.18) and 19 control patients (male: 9; female: 10; mean age: 24.65 ± 4.73) were included in the present study (Table 1).

**Table 1:** Main demographic and clinical data of patients and control subjects.

	OA-Group	Control-Group	*p*
Patients (n)	24	19	
Age			
Mean ± SD	68.19 ± 6.18	24.65 ± 4.73	0.001 *
Range	54–80	19–34	-
Gender			
Male, n (%)	11 (45.83%)	9 (47.37%)	0.221
Female, n (%)	13 (54.17%)	10 (52.63%)	0.124
BMI (kg/m^2^)			
Mean ± SD	27.75 ± 4.88	22.16 ± 1.7	0.002 *
Smoking status			
Number of smokers, n (%)	6 (20.69%)	2 (12.5%)	0.03 *
Kellgren Lawrence classification			
Grade 3	11 (37–93%)	//	//
Grade 4	18 (62.07%)	//	//
CRP (mg/dL)			
Mean ± SD	1.73 ± 1.25	0.21 ± 0.43	0.001 *
Serum Fibrinogen (mg/dL)			
Mean ± SD	302.4 ± 78.5	245.6 ± 57.3	0.003 *
KSS			
Mean ± SD	42.09 ± 7.24	94.45 ± 4.35	<0.001 *
IKDC			
Mean ± SD	0.346 ± 0.0825	0.964 ± 0.021	<0.001 *
KOOS main outcome			
Mean ± SD	38.45 ± 7.6	95.7 ± 3.5	<0.001 *
VAS			
Mean ± SD	5.78 ± 1.45	4.53 ± 1.65	0.07

* = significant *p*-value. BMI = Body Mass Index; IKDC = International Knee Documentation Committee; KSS = Knee Society Clinical Rating System; KOOS = Knee Injury and Osteoarthritis Outcome Score; VAS = Visual Analogue Scale for Pain; CRP = C-Reactive Protein.

The overall number of spots detected in the gel was similar between the two classes (OA vs. CTRL); indeed, image analysis showed 538 ± 51 for OA (coefficient of variation, CV, inter-assay 19.2%) and 611 ± 70 (CV 17.6%) for the control group.

**Figure 1:**
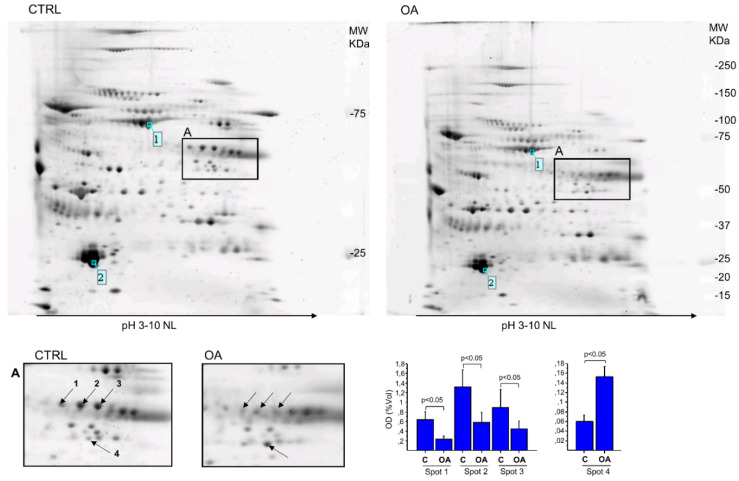
Two-dimensional gel electrophoresis (2DE) of SF sample. The representative 2D gel of enriched SF of OA and control (CTRL) patients are just representative images of the two gel classes, OA and CTRL. For each pooled synovial sample, a reference gel was made by combining all spots present in the replicate gels into one image. The reference maps of all samples belonging to the same population (i.e., CTRL or OA) formed a class. The semiquantitative variation in protein expression of each spot was measured by densitometric analysis of spots considering the fold change of the relative volumes (vol%) of spots in the two different classes. Only the statistically significant differences in the relative volumes (vol%) of spots in the two classes were reported here (arrows 1–4). The ImageMaster 2D Platinum analysis of SYPRO ruby-stained analytical 2-DE gels detected 611 ± 70 (mean ± SD) protein spots in CTRL and 538 ± 51 protein spots in OA patients. The area with the most relevant differences in protein expression among groups is evidenced by box A. At the bottom, on the right of the figure, the bar graph of the only protein spot differently expressed between the groups is reported. MW markers: Precision Plus dual colour. The light blue number indicates the reference protein spots shared (pairs) among all maps.

The gel’s image analysis allowed the quantification of only the spot intensities systematically present in all gels of each class. Four spots showed a significant level change between classes, including three spots down-expressed and one strongly over-expressed in OA groups compared to controls (Figure 1A).

The spots differentially expressed between the two classes were excised from the preparative gel and identified by mass spectrometry analysis, as reported in Table 2. Three spots (spots 1–3, Figure 1) exhibited a relevant downregulation (~70%) in the patient group and were identified as fibrinogen beta chain, a key constituent of the fibrin matrix (Table 2); one spot, in which levels of SF in OA patients were extremely increased compared to controls, was identified as alpha-enolase, a key enzyme involved in glycolysis as well as in several physiological processes such as cell growth control, tolerance to the effects of hypoxia, and allergic responses.

Table 3 shows the relationship between synovial fluid biomarkers, clinical scales, and serum biomarkers in OA patients.

**Figure 2:**
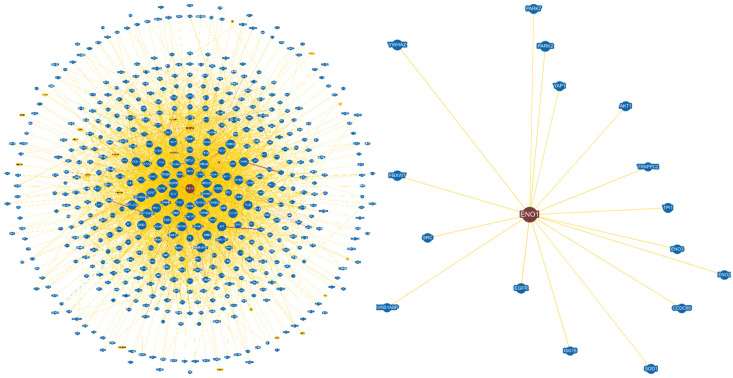
The network of protein-protein interactions of ENO1. The figure shows on the left the network of ENO1 interactions in BioGrids that have at least experimental evidence (as physical, genetic, and chemical interactions in small-scale or high-throughput experiments). The network of interactions between ENO1 and other proteins with at least three different and independent experimental evidence is reported on the right.

**Figure 3:**
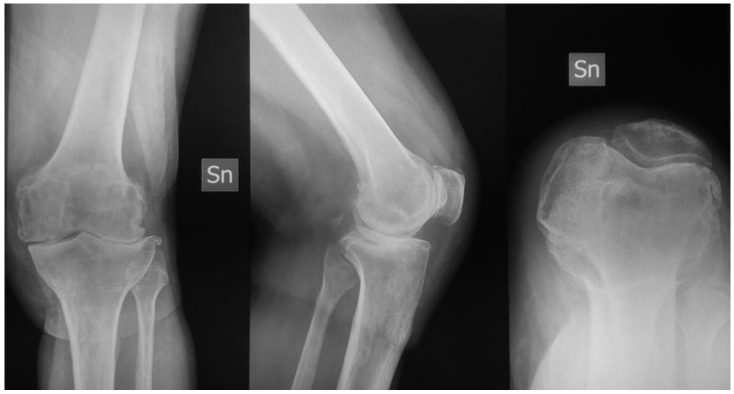
Preoperative X-rays of a 66 years-old woman undergoing TKR for knee osteoarthritis.

Figure 2 shows the network of protein-protein interactions of ENO1. Figure 3 shows the preoperative X-rays of Group-A patients affected by knee osteoarthritis.

### 3.2. Discussion

Osteoarthritis is a frequent age-related degenerative joint disease that causes loss in quality of life and functional decline [20,21,27]. It is a progressive, degenerative joint disease with a multi-factorial aetiology and could be considered the result of the interaction between both local and systemic factors [28].

Alpha-enolase (enolase-1 [ENO1]) is a multifunctional enzyme whose main role was historically identified in the glycolytic pathway; it is constitutively expressed in the cytosol of all cytotypes [29].

ENO1 could also be found on the cellular membrane, where it acts as a plasminogen-binding receptor, thus mediating the plasmin activation and extracellular matrix degradation.

This multifunctional glycolytic enzyme expressed abundantly in the cytosol is involved in the synthesis of pyruvate. Indeed, in the cytosol, it plays a central role in the second half of the glycolytic pathway, where it catalyzes the dehydration of 2-phospho-d-glycerate to phosphoenolpyruvate [29,30]. In the reverse reaction, i.e., the anabolic pathway known as gluconeogenesis, the enzyme catalyzes the hydration of phosphoenolpyruvate to 2-phospho-d-glycerate. In tumour cells, ENO1 is upregulated and supports anaerobic proliferation [29,30,31].

ENO1 is also expressed on the cellular membrane of several hematopoietic, epithelial, and endothelial cells, where it acts as a plasminogen receptor; therefore, it may play an important role in the intravascular and pericellular fibrinolytic system. ENO1 implication in systemic and invasive autoimmune disorders has been confirmed only in recent years [31].

Based on the analysis of the interactions using the BioGrid server [32], it is observed that ENO1 interacts with 519 different proteins, as reported in Figure 2, left panel. Focusing on those interactions with at least three pieces of independent experimental evidence, only a few proteins remain (Figure 2, right panel), which have often been associated in the literature with osteoarthritis. Particular attention should be given to the following proteins: the ubiquitin-like modifier ISG15 [22,23]; triosephosphate isomerase 1 (TPI1) [24]; Trafficking Protein Particle Complex Subunit 2 (TRAPPC2), which could lead to premature hip osteoarthritis, often requiring total hip replacement [25]; and Tyrosine 3-Monooxygenase/Tryptophan 5-Monooxygenase Activation Protein Zeta (YWHAZ), which has been considered as a candidate housekeeping gene in the synovium osteoarthritic knees [26]. Of particular interest is the interaction between ENO1 and EGFR, a receptor that in recent years has been associated with osteoarthritis [25,26,33,34,35,36,37,38,39,40,41,42,43,44,45].

Fibrinogen (FGB) is the precursor of fibrin, a protein that plays a central role in determining platelet aggregation and plasma viscosity [41]. It is also a biomarker of chronic inflammation [46]. It works as a messenger molecule and regulates the inflammatory response [23]. Recent studies have shown fibrinogen is a large plasma glycoprotein, present as a dimer, which is converted into a fibrin dimer by thrombin in long coagulation. Interestingly, fibrinogen and, therefore, fibrin have been found citrullinated in rheumatoid synovial tissue, suggesting their pathophysiological implications in rheumatoid arthritis [47].

Citrullinated proteins have been observed in all synovial exosome fractions [42,43]. Despite fibrinogen concentration being more strongly associated with poorer physical functioning among men than women, there were no statistically significant interaction effects between inflammatory biomarkers and sex [7].

Furthermore, it has been shown that fibrinogen was reduced after weight loss in patients with knee OA. Similarly, fibrinogen levels were reduced after supplementation of calcium fructoborate in patients with knee OA. Antony et al. [42] found that fibrinogen measured five years prior was negatively associated with tibial cartilage volume, but there was no significant association between the CRP (C-reactive protein) level and tibial cartilage volume in younger adults. These findings suggest that specific types of inflammation are associated with reduced “peak” knee cartilage volume in young adults. The association of fibrinogen and cartilage volume became of borderline significance after including fat mass in the model, indicating that this association is in part mediated by fat mass [46].

However, fibrinogen reduced the effect size of the sex difference by 37%, and sex explained only 0.9% of the variation in tibial cartilage volume after adjustment for fibrinogen [48].

Therefore, factors such as body composition, sex hormones, and fibrinogen correlate with knee cartilage volume in young adult life. In addition, the sex difference in knee cartilage volume is contributed largely by variations in body composition and/or fibrinogen [42,43,44,45].

Another important aspect is that fibrinogen levels were found to be lower after treatment with Pycnogenol than the initial values. In contrast, no significant changes for plasma free radicals, CRP (C-reactive protein), and fibrinogen were found in the placebo-treated group. Following treatment of osteoarthritis symptoms over three months, Pycnogenol lowered plasma free radicals, CRP levels, and fibrinogen values. CRP (C-reactive protein) levels were decreased by 71.3%, plasma free radicals by 29.9%, and fibrinogen by 37.1%, respectively. In contrast, treatment with placebo had only marginal and non-significant effects on all three parameters [46,47,48,49].

Current HA-based viscosupplementation provides only short-term relief in most cases due to instability and the relatively fast decomposition of the injected components [17,18,19,48,49,50,51,52,53,54]. To stabilize HA and prolong its intra-articular effect, two self-settling viscosupplementation compounds have been developed: RegenoGel and RegenoGel-OSP, in which high molecular weight HA is uniquely conjugated to purified or autologous plasma-derived fibrinogen, respectively [48,49].

In the present study, a comparative analysis of synovial fluid proteome was performed in patients with late-stage knee osteoarthritis compared to patients younger than 35 undergoing arthroscopic surgery for traumatic meniscus injuries. All of the control patients had no signs of early knee osteoarthritis at recruitment and presented only traumatic meniscus injuries. Patients with degenerative meniscus injuries were not included in the present study. Although synovial inflammation could also be detected in patients with meniscal tears [51], this study focuses on OA-related changes in the synovial fluid proteome; thus, the choice of the control group does not affect the study results.

Furthermore, even if meniscal tears could be a risk factor for knee OA [52,53], it should be noted that the control patients recruited in the present study had an acute traumatic meniscal injury with no signs of knee OA.

In the current study, we observed that the synovial fluid concentration of FGB is significantly lower in OA patients than in control patients. Interestingly, although the serum fibrinogen concentration is higher in OA patients than in control patients (Table 1), the synovial fluid analysis of OA patients depicted FGB downregulation in OA patients compared to non-OA patients. Moreover, a significant negative correlation between synovial mean FGB concentration and serum fibrinogen concentration (*p* = 0.001) was observed in the OA group. These findings could be explained considering a potential FGB consumption or structural modification in synovial fluid in patients with advanced knee OA (KL 3 or 4).

A negative correlation between synovial fluid mean FGB concentration and both KSS mean score (*p* = 0.02) and VAS mean score (*p* = 0.001) was also observed in OA patients. Hence, the synovial FGB downregulation correlates with patients’ pain levels and functional limitations.

In the present study, upregulation of ENO1 synovial concentration was also observed in OA patients compared to non-OA patients (Table 2). Moreover, in OA patients, a positive correlation was found between mean synovial ENO1 concentration and clinical scales (KOOS main outcome: *p* = 0.01; VAS = 0.005), thus highlighting a relationship between ENO1 upregulation and knee OA severity in the recruited patients.

The present study’s findings could be useful in further understanding OA pathogenesis and developing novel personalized therapies based on selective biomarkers modulation [45,46,47,48,49,50] to hopefully prevent OA progression and TKA surgery.

The main limitation of the present study is the small sample size, but rigid inclusion and exclusion criteria were used at recruitment. The pooled analysis of OA patients’ SF samples is another limitation of the study.

## 4. Conclusions

This study shows synovial fluid FBG downregulation and ENO1 upregulation could be useful biomarkers in diagnosing knee osteoarthritis. In the present study, we have also investigated the relationship between synovial biomarkers, serum biomarkers level, and clinical scales, thus highlighting a relationship between functional limitations and biomarkers concentration. We are aware that our findings need to be confirmed in a larger cohort. Further validation in a longitudinal study could also be useful to test these biomarkers in predicting the severity and evolution of knee OA.

## Figures and Tables

**Table 2:** Protein spots were differently expressed (*p* < 0.05, Mann-Whitney U test) in OA synovial fluid compared to control (CTRL) identified by mass spectrometry analysis.

Spot N°	Protein Name	Gene Name	Accession Number *	Theoretical MW (kDa)/pI	Experimental MW (kDa)/pI	Mascot Score	SC %	Unique Peptide	Fold Change (%)
									OA/CTRL
1	Fibrinogen beta chain	*FGB*	P02675	56.7/8.54	~57/8.8	106	25	13	0.37 (−63%)
2	Fibrinogen beta chain	*FGB*	P02675	56.7/8.54	~57/9.0	105	34	15	0.30 (−70%)
3	Fibrinogen beta chain	*FGB*	P02675	56.7/8.54	~57/9.2	67	25	12	0.30 (−70%)
4	Alpha-enolase	ENO1	P06733	47.4/7.01	~48/8.8	74	31	11	2.5 (+153%)

* Accession number in the UniProt database; Mascot score obtained from MS ion search against Swissprot [the Mascot score for an MS match is based on the absolute probability (P) that the observed match between the experimental data and the database sequence is a random event, the reported score is −10 Log(P), and the significance threshold is *p* < 0.05; all the ion scores are higher than the threshold values (see also www.matrixscience.com, accessed on 10 February 2023)]. The ratio between the mean value of spot intensity (vol%); SC: sequence coverage.

**Table 3:** Relationship between synovial FGB and ENO1 concentrations, clinical scales, and serum biomarkers in OA patients.

	FGB		ENO1	
	R	*p*	R	*p*
Clinical scales				
KSS	−0.61	0.02 *	0.33	0.108
IKDC	−0.32	0.12	0.43	0.09
KOOS main outcome	−0.44	0.08	0.68	0.01 *
VAS	−0.76	0.001 *	0.72	0.005 *
Serum biomarkers				
CRP	−0.23	0.45	0.27	0.32
Fibrinogen	−0.77	0.001 *	0.756	0.001 *

* significant *p*-value.

## Data Availability

Data available on request.

## Appendix A

Endonuclease IV *E. coli*, HALT protease phosphatase inhibitor cocktail, Agarose, RIPA buffer, Bis-Tris NUPAGE 4–12% Gel, MES SDS Running Buffer, SYPRO^®^ Ruby stain, Pierce C18 tips, and all reagents for protein in-gel tryptic digestion were from Thermo Fisher Scientific (Waltham, MA, USA); Colloidal Coomassie, proteins marker, and Bio-Radd Protein Assay were from Bio-Rad Laboratories (Hercules, CA, USA). Immobiline^®^ Drystrip pH 3–10, IPG Buffer pH 3–10, DeStreak Reagent, and glycerol were purchased from GEHealthcaree (Uppsala, Sweden). Hyaluronidase and all other chemicals were purchased from Sigma (Sigma Aldrich, St. Louis, MO, USA) unless otherwise mentioned. All the solvents used were Ultra-Resi-Analyzed grade.
